# Supplementary material for: Charting host-microbe co-metabolism in skin aging and application to metagenomics data
Source: PLoS One. 2021 Nov 10;16(11):e0258960. doi: 10.1371/journal.pone.0258960 (PMC8580226; doi:10.1371/journal.pone.0258960)
Supplement: S2 Table — (DOCX) [file pone.0258960.s002.docx]

**S2 Table. Significant relationship between SA scores and pathway level scores in skin swab samples of ‘young’ and ‘old’ subjects.** For each pathway and age group, a linear model was created for the relation between the gene hits score for a pathway (P_i_) and the SA score (SA):

$$SA=\beta_{0}+\beta_{1}P_{i}$$

in which β_0_ is the intercept term and β_1_ the regression coefficient. The linear models were implemented in R using the lm() function, and the raw P values for the β_1_ coefficient for each pathway were calculated. All P values were subsequently adjusted per age group, for multiple testing via the Holm method. Adjusted significant P values <0.05 are indicated in bold in the table. The R-script and metadata table used for this analysis has been made available in a [Github repository](https://github.com/andreiprodan/mask-publication).

|  | ‘Young’ | | ‘Old’ | |
| --- | --- | --- | --- | --- |
| **Pathway** | **Pval** | **Pval-adjust** | **Pval** | **Pval-adjust** |
| AcetylCoA biosynthesis | 0.036 | 0.164 | 0.484 | 1 |
| Protein glycation | 0.210 | 0.210 | 0.113 | 1 |
| Bacteriocins | 0.009 | 0.085 | 0.728 | 1 |
| Ceramide metabolism | 0.013 | 0.105 | 0.172 | 1 |
| Fatty acid oxidation | 0.052 | 0.164 | 0.748 | 1 |
| Fatty acid biosynthesis | 0.004 | **0.041** | 0.164 | 1 |
| Histidine metabolism | 0.003 | **0.033** | 0.187 | 1 |
| LTA synthesis | 0.031 | 0.164 | 0.467 | 1 |
| Pigmentation | 0.022 | 0.153 | 0.806 | 1 |
| Proteolytic activity | 0.012 | 0.105 | 0.106 | 1 |
| Radical protection | 0.002 | **0.024** | 0.023 | 0.303 |
| Skin penetration | 0.027 | 0.164 | 0.455 | 1 |
| Zinc sequestration | 0.027 | 0.164 | 0.455 | 1 |
