# Supplementary material for: Charting host-microbe co-metabolism in skin aging and application to metagenomics data
Source: PLoS One. 2021 Nov 10;16(11):e0258960. doi: 10.1371/journal.pone.0258960 (PMC8580226; doi:10.1371/journal.pone.0258960)
Supplement: S1 Appendix — Detailed description of materials and methods applied for the generation of a 16S rRNA gene dataset on the microbiota of cheek skin samples. (PDF) [file pone.0258960.s003.pdf]

S1 Appendix: Detailed description of materials and methods applied for generation of a 16S dataset on the microbiota of cheek skin samples

Facial skin sample collection of female volunteers

A human trial was performed for single sample collection of cheek samples from two groups of female subjects from the general European descent population (n=25 per group) in Belgium. The subject age was between 20 and 28 years (younger population group), and between 59 and 68 years (older population group). Exclusion criteria were: nodulo-cystic lesions/acne or sebaceous gland condition; eczema, psoriasis, atopy; prescribed or unprescribed use of skin treatment within 1 month prior to inclusion (oral or topical antibiotic, antifungal or topical steroids); smokers or having smoked in the past 2 years; recent history of chronic alcohol consumption defined as more than 15 standard servings per week or more than 3 servings per day; tanning bed usage less than 1 month prior to inclusion; sunbathing 1 month prior to inclusion; habitual exposure to sun or use of a tanning bed; clear UV light effects in the younger subjects that might be related to aging (inspected by the principal investigator during the recruitment procedure); primary immunodeficiency patients known to have dysbiosis in community diversity; use of tanning dihydroxyacetone (DHA) less than 1 month prior to study start; excessive habitual caffeine use (more than 6 small/medium cups of coffee or soda daily); body mass index higher than 30; pregnancy or lactation. At the moment of inclusion, subjects were requested to: refrain from using OTC products for any kind of skin treatment 7 days prior to the sampling; refrain from any facial

creams and make-up on the day of the sampling; refrain from swimming in a chlorinated pool, using a hot tub, sauna/steam baths, 48 hours prior to sampling visit; refrain from any facial treatments, facial masks, scrubs/peelings 2 weeks prior to the sampling visit; to follow specific bath/shower procedures (showering with plain water only, soap should be avoided as much as possible, and no scrubbing of the skin with a towel) during 24 hours prior to sampling visit – on the day of sampling showering was avoided completely (also with plain water).

The study was conducted according to the principles of the Declaration of Helsinki latest version Fortaleza, Brazil, October 2013. The candidates were informed verbally on the aim of the study and the study procedures. All participating subject signed the informed consent form. Data was analyzed anonymously. No IRB approval was requested for this study, for two reasons. Firstly, the study was carried out in Belgium, where IRB approval for this type of studies is not required. Secondly, there was no intervention or invasive procedure involved for the collection of the skin swab samples. Participants were requested to fill out a questionnaire (not considered as a psychological burden), and were only subjected to mild skin characteristics measurements.

During site visit information on the following parameters/metadata was collected: skin hydration (Corneometer, Courage&Khazaka); transepidermal water loss (TEWL) (Aquaflux, Biox); skin pH (Metrhom); skin smoothness, scaliness, sebum spots (Visioscan, Courage&Khazaka); standardized images were taken from the face (frontal and left view) with the Visia-CR (Canfield) to assess invisible and visible spots, red features, pores, porphyrins, color, fine lines, wrinkles and roughness. Additional metadata on skin properties was collected during the study by means of a questionnaire.

Samples were collected by means of swab sampling from the intact cheek skin. In short, skin sampling was performed using a standard custom-made sampling template, which allows for consistent sampling of 2 cm^2 of the cheek areas (both sides used). Before sampling of each subject, the template was cleaned with ethanol (70%) and air dried. To further minimize sample cross-contamination, a fresh pair of sterile gloves was worn by the person sampling each individual. The area within the template was swabbed with a sterile HydraFlock collection swab (3206H-25; Puritan Diagnostics, USA).

A sterile HydraFlock swab (3206H-25; Puritan Diagnostics, USA) was soaked in sterile PBS+ solution (PBS of pH 7.0 with 0.5% Tween-20). After soaking in PBS+ solution, the swab was dried very briefly on a sterile gauze before the start of the sampling. Samples were taken by direct swabbing of the cheek skin. The shaft of the swab was held parallel to the skin surface and it was rubbed back and forth 10 times applying firm pressure. Immediately after swabbing, each swab was swirled in a 1.5 ml collection tube with 0.25 ml of sterile PBS+ solution with 2.5 µl of 1 mM EDTA (DNase inhibitor). The swab was left in the solution and the samples were stored on ice before freezing and shipment on dry ice for further processing at NIZO.

Bacterial DNA extraction, PCR amplification and 16S rRNA gene Illumina sequencing

The skin swabs in PBS+ solution were thawed and the swabs were placed into DNA IQ Spin baskets (Promega) and centrifuged for 1 min at 10,621g. to collect the liquid/material that was still in the swabs. The resulting liquid was combined with the PBS+ solution and DNA was isolated

using the DNeasy UltraClean Microbial DNA isolation kit (Qiagen) according to the manufacturer's instructions. To the combined PBS+ solution 250 µl Powerbead solution and 50 µl SL was added. The mixture was incubated at 70°C for 10 min. Next steps were performed according to the manufacturer's instructions and DNA was eluted in 30 µl EB buffer.

Using a 2-step PCR, barcoded amplicons from the V3–V4 region of 16S rRNA genes were generated. For initial amplification of the V3–V4 part of the 16S rRNA universal primers were used with the following sequences: forward primer, '5-*TCGTCGGCAGCGTCAGATGTGTATAAGAGACAG***<u>CCTACGGGAGGCAGCAG'</u>** (broadly conserved bacterial primer 357F in bold and underlined); reverse primer, '5-*GTCTCGTGGGCTCGGAGATGTGTATAAGAGACAG***<u>TACNVGGGTATCTAAKCC</u>**' (broadly conserved bacterial primer 802R (with adaptations) in bold and underlined), appended with Illumina adaptor sequences (in italics). The PCR amplification mixture contained: 6 µL skin sample DNA, and 14 µL master mix (0.2 µL Phusion DNA Polymerase (2 U/µL; ThermoFisher Scientific, Waltham, MA, USA), 4 µL Phusion HF buffer (5×), 0.4 µL dNTP mix (10 mM each)), 0.1 µL (100 µM) of forward primer, 0.1 µL (100 µM) of reverse primer and 8.6 µL sterile water (total volume 20 µL). PCR conditions were: 95 °C for 2 min, followed by 33 cycles of 95 °C for 20 sec, 55 °C for 20 sec and 72 °C for 1 min. This was followed by a final extension for 10 min at 72 °C. We then purified the approximately 500 bp PCR amplicons using the MSB Spin PCRapace kit (Invitek Molecular, Berlin, Germany).

For the library PCR step (step 2) in combination with sample-specific barcoded primers, purified PCR products were shipped to BaseClear BV (Leiden, The Netherlands). PCR products were purified, checked on a Bioanalyzer (Agilent) and quantified. This was followed by multiplexing,

clustering and sequencing on an Illumina MiSeq with the paired-end (2x) 300 bp protocol and indexing. FASTQ read sequence files were generated using bcl2fastq2 version 2.18. Initial quality assessment was based on data passing the Illumina Chastity filtering. From the raw sequencing data, the sequence reads of too low quality (only "passing filter" reads were selected) were discarded and reads containing adaptor sequences or PhiX control were removed. On the remaining reads a quality assessment was performed using the FASTQC tool version 0.11.5.

16S rRNA gene sequence analysis and statistics

16S rRNA gene sequence pairs were assembled into pseudoreads with PEAR, using default settings, and were analyzed using a workflow based on Qiime 1.8 [1]. We performed operational taxonomic unit (OTU) clustering (open reference), taxonomic assignment and reference alignment with the pick_open_reference_otus.py workflow script of Qiime, using uclust as clustering method (97% identity) and GreenGenes v13.8 as reference database for taxonomic assignment. Reference-based chimera removal was done with Uchime [2]. The RDP classifier version 2.2 was performed for taxonomic classification [3]. Statistical tests were performed as implemented in SciPy (https://www.scipy.org/), downstream of the Qiime-based workflow.

Between-group differences of single taxa were assessed using the non-parametric Mann-Whitney U test with FDR correction for multiple testing; unless stated otherwise. For comparisons of more than 2 groups the non-parametric Kruskal-Wallis test with Dunn’s posthoc test was applied, as implemented in Graphpad Prism 5.01.

We performed multivariate redundancy analyses (RDAs) on the gut microbiota composition as assessed by 16S rRNA gene sequencing in Canoco version 5.11 using default settings of the analysis type “Constrained” [4]. Relative abundance values of genera or OTUs were used as

response data, and metadata as explanatory variable. For visualization purposes, genera (and not OTUs) were plotted as supplementary variables. RDA calculates p-values by permutating (Monte Carlo) the sample status. In all analyses, p values < 0.1 were considered statistical trends; p values < 0.05 were considered statistically significant.

Microbial functionalities analysis based on 16S rRNA gene sequences

In order to perform a more dedicated analysis than provided by the generic PICRUSt algorithm, specific microbial pathways were identified on basis of the scientific literature. These were selected as follows. First a literature search was performed to identify the key host molecular pathways that are potentially involved in skin aging. Subsequently, the metabolites and signaling molecules that play a role in these pathways were identified and matched to known molecular pathways in skin microorganisms. In case of matching pathways, the microbial genes in these pathways were identified. In short, a set of bacterial reference genomes was chosen, based on relevant bacterial taxa identified as described above, supplemented with the highly abundant taxa in the samples taken in this project as determined through 16S amplicon sequencing. The evolutionary relation between all genes in this reference set was reconstructed using Orthagogue [5]. Combined with the specific genes linked to skin aging described above, this provided us with a matrix outlining the presence - absence profiles in these reference organisms of the skin aging-related bacterial genes. This presence - absence data was linked to the compositional data by including 16S fragments of the reference gnomes in the NIZO 16S analysis workflow, resulting in a predicted inferred functionality that is similar to the output of PICRUSt, but specifically focused

on the samples taken in this project and our research questions. Where applicable, statistical analysis of functionality data was performed in the same way as for the compositional data.

Skin Aging (SA) Score

In the analysis of the relation between skin parameters and microbiota composition, multiple parameters describing skin characteristics (pores number, roughness, wrinkles number, porphyrins number, red features number, skin color evenness, spots visible number and spots invisible number) were combined into a single "Skin Aging" parameter by taking the average of the normalized parameter values. Min-Max normalization was performed in the format "normalized_value = (measured_value - lowest value) / (highest_value - lowest_value)".

References

1. Caporaso JG, Kuczynski J, Stombaugh J, Bittinger K, Bushman FD, et al. (2010) QIIME allows analysis of high-throughput community sequencing data. Nat Methods 7: 335-336.
2. Edgar RC, Haas BJ, Clemente JC, Quince C, Knight R (2011) UCHIME improves sensitivity and speed of chimera detection. Bioinformatics 27: 2194-2200.
3. Cole JR, Wang Q, Cardenas E, Fish J, Chai B, et al. (2009) The Ribosomal Database Project: improved alignments and new tools for rRNA analysis. Nucleic Acids Res 37: D141-145.
4. Braak CJFt, Šmilauer P (2012) CANOCO reference manual and user's guide : software for ordination (version 5.0). Wageningen etc.: Microcomputer power, Itaca. 496 p p.
5. Ekseth OK, Kuiper M, Mironov V (2014) orthAgogue: an agile tool for the rapid prediction of orthology relations. Bioinformatics 30: 734-736.
