# Supplementary material for: Charting host-microbe co-metabolism in skin aging and application to metagenomics data
Source: PLoS One. 2021 Nov 10;16(11):e0258960. doi: 10.1371/journal.pone.0258960 (PMC8580226; doi:10.1371/journal.pone.0258960)
Supplement: S2 Appendix — Detailed description of co-metabolic processes in human cells that are involved in skin aging with links to microbial functionalities in the skin microbiome. (PDF) [file pone.0258960.s004.pdf]

S2 Appendix : Detailed description of co-metabolic processes in human cells that are involved in skin aging with links to microbial functionalities in the skin microbiome.

UV-B induced immune suppression

Urocanic acid is a deamination product of histidine. It is a compound that is involved in and mediates UVB-induced immune suppression (1, 2) and has also been shown to suppress contact hypersensitivity in mice. The mechanism by which urocanic acid (via its cis-isomer, which is formed by photoisomerization from its trans-isomer in the stratum corneum) performs this action is not fully understood, but might involve binding to histamine or histamine-like receptors and GABA receptors (3).

Bacterial link

Trans-urocanic acid is produced from histidine via the action of L-histidine ammonia lyase (hal). In microorganisms (e.g. *Corynebacterium resistens* DSM 41500), the genes for histidine metabolism are organized in the Hut (histidine utilisation) cluster, where *hutH* is responsible for the conversion of histidine to urocanic acid (4, 5). Specific cis-urocanic acid degrading activity has also been demonstrated in *Micrococcus luteus* (6, 7).

Histidine metabolism / NMF production

Histidine and its breakdown products are natural moisturizers of the skin. Oral supplementation of histidine was shown to be effective in atopic dermatitis patients (8, 9). A major source of histidine and related products is the breakdown of filaggrin (FLG). The breakdown products do not only play a role in moisturizing, they also influence directly the protein expression of skin organisms such as *Staphylococcus aureus* (10, 11). Also, other amino acids can serve as natural moisturizers. The largest component (~40%) of Natural Moisturising Factors (NMF) is the group of free amino acids. Of these, L-serine is the most abundant (~36%) followed by glycine (22%) and L-alanine (13%). Histidine, ornithine, citrulline and arginine all account for 6–8% of free amino acids within NMF (12).

Bacterial link

Histidine conversion in microorganisms can occur via the Hut (histidine utilization) pathway in which L-histidine is converted to L-glutamate. The operon consists of *hutH, hutU, hutI* and *hutG* genes as well as the regulatory gene *hutR*. In Gram-negative organisms, histidine conversion is controlled by the transcriptional repressor *hutC* (13, 14). An increased activity in this pathway could lead to reduction in skin hydration.

Protein glycation

One of the mechanisms of skin aging is believed to be glycation of proteins (15). Protein glycation is a complicated process that involves the reversible production of Amadori products by the

reaction of the carbonyl functionalities of reducing sugars with the free amino groups of proteins to form ketoamine intermediates, also called Amadori products. These Amadori products can either be removed by the cells or irreversible react with amino acids of peptide residues and proteins to form protein adducts and protein cross links. The main target of protein glycation in the skin is collagen. Collagen glycation leads to reduced elasticity of the skin and to wrinkle formation.

Bacterial link

Removal of the Amadori products can be accomplished by the action of glutathione-dependent glyoxalases that convert glyoxal, methylglyoxal and other alpha-oxoaldehydes to d-lactate. Other enzymes that convert Amadori products are fructosyl amine oxidases (aka amadoriases), which are solely expressed in bacteria and yeast, but not in mammals (16) and that act on low molecular weight Amadori derivatives. Another class of enzymes are the fructosamine kinases that destabilize Amadori products by phosphorylation, which are (in contrast to the fructosyl amine oxidases) also expressed in humans. The conversion of Amadori products by bacteria has not yet been experimentally demonstrated.

Pigmentation

Melanine is produced from L-phenylalanine / tyrosine via a series of steps involving the intermediates DOPA and DOPAquinone (via the Raper-Mason patwhay) (17). DOPAquinone is further converted to dopachrome Eu-melanins. Reaction of DOPAQuinone with Glutathione or Cysteine leads to the formation of Pheo-melanins. The first committed steps (Tyrosine to

DOPAQuinone) are catalyzed by tyrosinase (EC 1.14.18.1, also referred to as tyrosine hydroxylase), which is a copper-containing monooxygenase catalyzing the o-hydroxylation of monophenols to the corresponding catechols (monophenolase or cresolase activity) and the oxidation of monophenols to the corresponding o-quinones (diphenolase or catecholase activity). Kojic acid, which is an inhibitor of this enzyme, is used as a skin whitening cosmetic agent. Tyrosinase (TYR) catalyzes the initial rate-limiting step in melanogenesis, the hydroxylation of tyrosine to b-3,4-dihy- droxyphenylalanine (DOPA) and the subsequent oxidation of DOPA to DOPAquinone. Tyrosinase is responsible for the critical rate-limiting steps of melanogenesis; mutations that affect TYR function result in albinism.

Bacterial link

Bacterial tyrosinases have been described in literature, but have not yet been linked to skin organisms (18-20). One could also imagine that general L-amino acid oxidases with a broad substrate specificity could perform this action, for instance as has been described for *Mycobacterium leprae* (21).

C2 ceramides are able to inhibit the function of tyrosinase and related enzymes (TYRP1 and DCT), mainly through down-regulation of MITF. Hypopigmenting properties of unsaturated fatty acids (e.g. linoleic acid) result from increased ubiquitination of tyrosinase that decreases its enzymatic function.

Fatty acid metabolism

The strateum corneum is an essential part of the skin barrier, consisting of terminally differentiated keratinocytes and corneocytes, embedded in a lipid-rich extracellular matrix. The breakdown of this barrier is one of the features of skin aging. The strateum corneum matrix consists of extracellular lipids, composed of among others ceramides, cholesterol and free fatty acids. Ceramides and sphingosine are synthesized from the free fatty acids, most notably palmitoleate (22, 23). It has been well documented that dysregulation of the fatty acid composition in the skin or the deficiency of essential fatty acids has a significant effect on the barrier function of the skin (24-28).

Bacterial link

Fatty acids are essential parts of bacterial membranes. Fatty acid metabolism in bacteria is composed of two important pathways. Important gene clusters in these pathways are the *fab* operon for the synthesis of fatty acids, (consisting of *ACC*, *fabD*, *fabH*, *fabF/fabB*, *fabG*, *fabZ* and *fabI*), and the *fad* operon involved in the degradation of fatty acids via the b-oxidation pathway (including *fadL*, *fadD*, *fadE*, *fadH* and *fadA*). Members of these operons have been well studied for skin organisms (29-32).

Ceramide and sphingosine metabolism

The upper stratum corneum contains a number of ceramide molecules. Incorrect ceramide metabolism has been associated with a deceased barrier function and is associated with the occurrence of atopic dermatitis and wrinkle formation. Ceramide is synthesized from palmitoyl-CoA and serine and serves as a precursor for the formation of sphingomyelin and glucocerebroside.

Deacylases can convert sphingomyelin and glucocerebroside to sphingosylphosphoryl choline and glucosylceramide respectively, with the concomitant production of free fatty acids.

Bacterial link

Bacterial ceramidases (EC 3.5.1.23) are amidohydrolases that hydrolyse the amide bond in ceramides to yield free fatty acid and sphingosine. In *Pseudomonas aeruginosa* this enzyme is under the control of a transcriptional regulator, *SphR*, a putative araC family transcriptional regulator (33), which in turn is regulated / bound by free sphingoid bases such as sphingosine, dihydrosphingosine, and phytosphingosine, but not sphingomyelin or ceramide. Complete ceramide synthesizing pathways have not been described in bacteria.

Porphyrin synthesis

Porphyrins are molecules with a number of immunological functions. Overproduction of porphyrins may trigger inflammation and has been (amongst others) implicated in the development of acne (34, 35).

Bacterial link

Porphyrins are synthesized from L-glutamate via genes located in the *Hem* operon. In *Propionibacterium acnes* it has been shown that suppression of Vitamin B12 synthesis from L-glutamate (e.g. by the addition of external Vitamin B12) directs the L-glutamate towards porphyrin synthesis (34, 36).

It has also been described that coproporphyrin produced by *Cutibacterium* spp. (formerly known as *Propionibacterium* spp.) induced aggregation of *S. aureus*, thus indicating a role for interspecies communication for these molecules (25053784). This operon has also been described for *S. aureus* (37), *Veillonella* (38) and *Propionibacterium freudenreichii* (39).

Oxygen radical production and scavenging

A large contributor to skin aging is the production of free oxygen radicals (ROS). One of the major causes of skin aging is believed to be the reduced capacity of the skin to defend itself against these radicals. Radical production may be induced by conversion of purines (adenosine) to uric acid via hypoxanthine (40-42).

Furthermore, air pollutants are known to contribute to the production of ROS. It has been described that polycyclic aromatic hydrocarbons (PAHs), such as benzo[a]pyrene, induce an inflammatory response in the skin via the aryl hydrocarbon receptor (AhR), resulting in the production of ROS. Free radicals are removed via the consecutive action of superoxide dismutase and catalase and via the reaction of H_2O_2 with glutathione, catalyzed by glutathione peroxidase.

Bacterial link

Bacterial production of extracellular Superoxide Dismutase (SOD) or catalase may prevent oxidative damage or UVB-induced damage to the skin. Other ROS scavengers are GSH (glutathione), produced from homocysteine. Cysteine is transformed to gamma-glutamylcysteine (via L-glutamate and cysteine through the enzymatic activity of gamma-glutamylcysteine synthetase (glutamate cysteine ligase, GCL)) and the subsequent addition of glycine to the C-

terminal of gamma-glutamylcysteine via the enzyme glutathione synthetase. Another route involves the addition of glycine to yield cystein-glycin, after which glutamate is added.

Bacterial glutathione peroxidase has been hypothesized to be also beneficial for the survival of pathogens on the skin, because it protects the bacteria against oxidative stress (43).

Proteolytic activity

The upper stratum corneum contains a number of protein crosslinks that form a barrier. Proteolysis of these cross links leads to a loss of barrier function. Proteases that are able to do this are in the human MMP and yy. Kallikreins and SPINK. Also the inhibitors like TIMP are important.

Bacterial link

Bacterial production of extracellular proteases has been reported to target vimentin, elastin and other structural proteins, and host immune molecules (44, 45). Some of these bacterial proteases also work against other bacteria. For example the protease LasA of *Pseudomonas aeruginosa* has been reported to show activity against *S. aureus*. *S. aureus* itself has been described to produce various proteases that can impact innate immune components or disrupt the extracellular matrix, such as SpIA and EpiP, as reviewed by Pietrocola and coworkers (46). Furthermore, *S. aureus* secretes proteins such as superantigen-like proteins SSL1 and SSL5, which can modulate metalloproteases present in the skin, or serine aspartate glycosyltransferases A and B, which can inhibit catepsin G that is produced by neutrophils as part of the innate immune system.

References

1. de Fine Olivarius F, Wulf HC, Crosby J, Norval M. Sunscreen protection against cis-urocanic acid production in human skin. Acta Derm Venereol. 1999;79(6):426-30. Epub 1999/12/22.

2. Gruner S, Oesterwitz H, Stoppe H, Henke W, Eckert R, Sonnichsen N. Cis-urocanic acid as a mediator of ultraviolet-light-induced immunosuppression. Semin Hematol. 1992;29(2):102-7. Epub 1992/04/01.

3. Uusi-Oukari M, Soini SL, Heikkila J, Koivisto A, Neuvonen K, Pasanen P, et al. Stereospecific modulation of GABA(A) receptor function by urocanic acid isomers. Eur J Pharmacol. 2000;400(1):11-7. Epub 2000/07/29.

4. Koizumi H, Shimizu T, Nishino H, Ohkawara A. Cis-urocanic acid attenuates histamine receptor-mediated activation of adenylate cyclase and increase in intracellular Ca2+. Arch Dermatol Res. 1998;290(5):264-9. Epub 1998/07/29.

5. Schroder J, Maus I, Ostermann AL, Kogler AC, Tauch A. Binding of the IclR-type regulator HutR in the histidine utilization (hut) gene cluster of the human pathogen Corynebacterium resistens DSM 45100. FEMS Microbiol Lett. 2012;331(2):136-43. Epub 2012/04/03.

6. Hug DH, Dunkerson DD, Hunter JK. The degradation of L-histidine and trans- and cis-urocanic acid by bacteria from skin and the role of bacterial cis-urocanic acid isomerase. J Photochem Photobiol B. 1999;50(1):66-73. Epub 1999/08/12.

7. Jack TR, Zajic JE. The enzymatic conversion of L-histidine to urocanic acid by whole cells of Micrococcus luteus immobilized on carbodiimide activated carboxymethylcellulose. Biotechnol Bioeng. 1977;19(5):631-48. Epub 1977/05/01.

8. Tan SP, Brown SB, Griffiths CE, Weller RB, Gibbs NK. Feeding filaggrin: effects of l-histidine supplementation in atopic dermatitis. Clin Cosmet Investig Dermatol. 2017;10:403-11. Epub 2017/10/19.

9. Zeeuwen PL, Ederveen TH, van der Krieken DA, Niehues H, Boekhorst J, Kezic S, et al. Gram-positive anaerobe cocci are underrepresented in the microbiome of filaggrin-deficient human skin. J Allergy Clin Immunol. 2017;139(4):1368-71. Epub 2016/10/12.

10. Miajlovic H, Fallon PG, Irvine AD, Foster TJ. Effect of filaggrin breakdown products on growth of and protein expression by Staphylococcus aureus. J Allergy Clin Immunol. 2010;126(6):1184-90 e3. Epub 2010/11/03.

11. Rippke F, Schreiner V, Doering T, Maibach HI. Stratum corneum pH in atopic dermatitis: impact on skin barrier function and colonization with Staphylococcus Aureus. Am J Clin Dermatol. 2004;5(4):217-23. Epub 2004/08/11.

12. Arezki NR, Williams AC, Cobb AJ, Brown MB. Design, synthesis and characterization of linear unnatural amino acids for skin moisturization. International journal of cosmetic science. 2017;39(1):72-82. Epub 2016/07/06.

13. Zhang XX, Rainey PB. Genetic analysis of the histidine utilization (hut) genes in Pseudomonas fluorescens SBW25. Genetics. 2007;176(4):2165-76. Epub 2007/08/25.

14. Zhang XX, Ritchie SR, Rainey PB. Urocanate as a potential signaling molecule for bacterial recognition of eukaryotic hosts. Cell Mol Life Sci. 2014;71(4):541-7. Epub 2013/12/07.

15. Gkogkolou P, Bohm M. Advanced glycation end products: Key players in skin aging? Dermatoendocrinol. 2012;4(3):259-70. Epub 2013/03/08.

16. Lin Z, Zheng J. Occurrence, characteristics, and applications of fructosyl amine oxidases (amadoriases). Appl Microbiol Biotechnol. 2010;86(6):1613-9. Epub 2010/03/30.

17. Prota G. Regulatory mechanisms of melanogenesis: beyond the tyrosinase concept. J Invest Dermatol. 1993;100(2 Suppl):156S-61S. Epub 1993/02/01.

18. Claus H, Decker H. Bacterial tyrosinases. Syst Appl Microbiol. 2006;29(1):3-14. Epub 2006/01/21.

19. Nikodinovic-Runic J, Martin LB, Babu R, Blau W, O'Connor KE. Characterization of melanin-overproducing transposon mutants of Pseudomonas putida F6. FEMS Microbiol Lett. 2009;298(2):174-83. Epub 2009/07/25.

20. Nolan LC, O'Connor KE. A spectrophotometric method for the quantification of an enzyme activity producing 4-substituted phenols: determination of toluene-4-monooxygenase activity. Anal Biochem. 2005;344(2):224-31. Epub 2005/08/03.

21. Prabhakaran K. Unusual effects of reducing agents on 0-diphenoloxidase of Mycobacterium leprae. J Bacteriol. 1971;107(3):787-9. Epub 1971/09/01.

22. Dumas SN, Ntambi JM. A Discussion on the Relationship between Skin Lipid Metabolism and Whole-Body Glucose and Lipid Metabolism: Systematic Review. Journal of cell signaling. 2018;3(3). Epub 2018/11/27.

23. Proksch E, Holleran WM, Menon GK, Elias PM, Feingold KR. Barrier function regulates epidermal lipid and DNA synthesis. The British journal of dermatology. 1993;128(5):473-82. Epub 1993/05/01.

24. Li W, Sandhoff R, Kono M, Zerfas P, Hoffmann V, Ding BC, et al. Depletion of ceramides with very long chain fatty acids causes defective skin permeability barrier function, and neonatal lethality in ELOVL4 deficient mice. International journal of biological sciences. 2007;3(2):120-8. Epub 2007/02/22.

25. Jungersted JM, Hellgren LI, Jemec GB, Agner T. Lipids and skin barrier function--a clinical perspective. Contact dermatitis. 2008;58(5):255-62. Epub 2008/04/18.

26. Downing DT, Wertz PW, Stewart ME. The role of sebum and epidermal lipids in the cosmetic properties of skin. International journal of cosmetic science. 1986;8(3):115-23. Epub 1986/06/01.

27. Feingold KR. Thematic review series: skin lipids. The role of epidermal lipids in cutaneous permeability barrier homeostasis. Journal of lipid research. 2007;48(12):2531-46. Epub 2007/09/18.

28. van Smeden J, Bouwstra JA. Stratum Corneum Lipids: Their Role for the Skin Barrier Function in Healthy Subjects and Atopic Dermatitis Patients. Current problems in dermatology. 2016;49:8-26. Epub 2016/02/06.

29. Barzantny H, Guttmann S, Lassig C, Brune I, Tauch A. Transcriptional control of lipid metabolism by the MarR-like regulator FamR and the global regulator GlxR in the lipophilic axilla isolate Corynebacterium jeikeium K411. Microbial biotechnology. 2013;6(2):118-30. Epub 2012/11/21.

30. Barzantny H, Brune I, Tauch A. Molecular basis of human body odour formation: insights deduced from corynebacterial genome sequences. International journal of cosmetic science. 2012;34(1):2-11. Epub 2011/07/28.

31. Schiebel J, Chang A, Merget B, Bommineni GR, Yu W, Spagnuolo LA, et al. An ordered water channel in Staphylococcus aureus FabI: unraveling the mechanism of substrate recognition and reduction. Biochemistry. 2015;54(10):1943-55. Epub 2015/02/24.

32. Chirgadze NY, Briggs SL, McAllister KA, Fischl AS, Zhao G. Crystal structure of Streptococcus pneumoniae acyl carrier protein synthase: an essential enzyme in bacterial fatty acid biosynthesis. The EMBO journal. 2000;19(20):5281-7. Epub 2000/10/18.

33. Okino N, Ito M. Molecular mechanism for sphingosine-induced Pseudomonas ceramidase expression through the transcriptional regulator SphR. Sci Rep. 2016;6:38797. Epub 2016/12/13.

34. Kang D, Shi B, Erfe MC, Craft N, Li H. Vitamin B12 modulates the transcriptome of the skin microbiota in acne pathogenesis. Sci Transl Med. 2015;7(293):293ra103. Epub 2015/06/26.

35. Pecastaings S, Roques C, Nocera T, Peraud C, Mengeaud V, Khammari A, et al. Characterisation of Cutibacterium acnes phylotypes in acne and in vivo exploratory evaluation of Myrtacine((R)). J Eur Acad Dermatol Venereol. 2018;32 Suppl 2:15-23. Epub 2018/06/13.

36. Barnard E, Johnson T, Ngo T, Arora U, Leuterio G, McDowell A, et al. Porphyrin Production and Regulation in Cutaneous Propionibacteria. mSphere. 2020;5(1). Epub 2020/01/17.

37. Choby JE, Grunenwald CM, Celis AI, Gerdes SY, DuBois JL, Skaar EP. Staphylococcus aureus HemX Modulates Glutamyl-tRNA Reductase Abundance To Regulate Heme Biosynthesis. mBio. 2018;9(1). Epub 2018/02/14.

38. Zhou P, Li X, Qi F. Identification and characterization of a haem biosynthesis locus in Veillonella. Microbiology. 2016;162(10):1735-43. Epub 2016/08/28.

39. Hashimoto Y, Yamashita M, Murooka Y. The Propionibacterium freudenreichii hemYHBXRL gene cluster, which encodes enzymes and a regulator involved in the biosynthetic pathway from glutamate to protoheme. Appl Microbiol Biotechnol. 1997;47(4):385-92. Epub 1997/04/01.

40. Heck DE, Shakarjian M, Kim HD, Laskin JD, Vetrano AM. Mechanisms of oxidant generation by catalase. Ann N Y Acad Sci. 2010;1203:120-5. Epub 2010/08/19.

41. Munoz-Munoz JL, Garcia-Molina F, Varon R, Tudela J, Garcia-Canovas F, Rodriguez-Lopez JN. Generation of hydrogen peroxide in the melanin biosynthesis pathway. Biochim Biophys Acta. 2009;1794(7):1017-29. Epub 2009/04/21.

42. Randhawa M, Sangar V, Tucker-Samaras S, Southall M. Metabolic signature of sun exposed skin suggests catabolic pathway overweighs anabolic pathway. PLoS One. 2014;9(3):e90367. Epub 2014/03/08.

43. Brenot A, King KY, Janowiak B, Griffith O, Caparon MG. Contribution of glutathione peroxidase to the virulence of Streptococcus pyogenes. Infect Immun. 2004;72(1):408-13. Epub 2003/12/23.

44. Koziel J, Potempa J. Protease-armed bacteria in the skin. Cell Tissue Res. 2013;351(2):325-37. Epub 2012/02/24.

45. Nowak D, Miedzobrodzki J. Serine proteinase from Staphylococcus aureus enhances elastin degradation by elastases in the presence of human alpha-1-proteinase inhibitor. Antonie Van Leeuwenhoek. 1991;59(2):109-14. Epub 1991/02/01.

46. Pietrocola G, Nobile G, Rindi S, Speziale P. Staphylococcus aureus Manipulates Innate Immunity through Own and Host-Expressed Proteases. Front Cell Infect Microbiol. 2017;7:166. Epub 2017/05/23.
