# Supplementary material for: Charting host-microbe co-metabolism in skin aging and application to metagenomics data
Source: PLoS One. 2021 Nov 10;16(11):e0258960. doi: 10.1371/journal.pone.0258960 (PMC8580226; doi:10.1371/journal.pone.0258960)
Supplement: Attachment — Submitted filename: Response to reviewers.docx [file pone.0258960.s008.docx]

**Point-to-point response to reviewers’ comments**

**Reviewer #1**

*1) More details on the various steps in data processing and analysis could be added. In particular, a schematic figure (or figures) summarizing the approach and experiments would help to more easily understand the overall study setup and put the various pieces in context. The Methods section should then be compared to such overview in order to make sure that the necessary technical details of each step are provided so that the research is reproducible*.
**We agree with the reviewer that this is indeed a complicated workflow and support the suggestion of a summarizing figure. We have now included a diagram (Figure 1) at the start of the Results section (lines 247 – 260 of the ‘revised Manuscript with Track Changes’ file) that describes the entire experimental process, starting from the literature study to identify the co-metabolic pathways to the generation of the Hidden Markov Models and the application of these HMMs to the various data types that were collected.
The HMMs were designed specifically for skin microorganisms (in contrast to already existing signatures in publicly available databases that are based on a generic and broad range of microorganisms), which limits the application of these models to this specific application. The description of the multistep approach is linked to the results obtained and described in the remainder of the Results section, by indicating reference to the other figures in the body text. It should be noted that this workflow does not constitute a design for a new software package; in most steps we are using existing, publicly available software, for application in this specific project.**

*2) I did not find mention of the source code availability. It is paramount that the code for the experiments including data processing, method, and comparisons, would be available for further verification, benchmarking, and development*.
**We have now included the flow of information in the project, which we believe is sufficient for people that are skilled in the art to reproduce this workflow (Figure 1). It should be noted that this workflow does not constitute a design for a new software package; in most steps we are using existing, publicly available software, for application in this specific project.**
**In addition, we emphasize that our analysis includes a number of manual steps (e.g., file handling, expert interpretation) that cannot be made available through source code. For example, during construction of the HMM models, a number of heuristics and expert calls were made to determine the exact cut-off score for the thresholds of the HMM score (now mentioned more explicitly in the M&M section, line 139). Our aim never was to provide a ready-to-use software package, and there is no workflow script available for that (also see the final sentence of the second paragraph in the Discussion section (lines 439 – 441 of the ‘revised Manuscript with Track Changes’ file): “*The process described in this paper uses a manual process for creating these HMMs based on manual curation of a set of scientific articles.*”)**

**We trust that the addition of the schematic figure on the multistep approach (Fig 1) and the additional explanation on manual curation sufficiently covers this reviewer’s comment.**

*3) The work would benefit from verification on another body site but I can see that this might be out of scope for this manuscript; it would be informative, however, to discuss in more length the possible limitations and challenges that one might encounter when extending the analysis to other body sites.*

**We agree with the reviewer that a statement on the generic nature of the method is in order. We have updated the Discussion section with the following paragraph (lines 466 – 472 of the ‘revised Manuscript with Track Changes’ file):**

**“This approach can also be applied on data derived from other body sites (e.g. gut, oral cavity, etc), although the set of reference organisms to include should be adapted to reflect relevance to that particular body site. In case of application of the approach on different site on skin, the same reference organisms can be used as described in this manuscript as these are a relevant representation of organisms that can be found at multiple sites on the skin.”**

**In this study, we focused specifically on skin samples from the cheek site, because of the research question related to aging skin, which is particularly relevant for the facial area.**

* Minor

*4) All data are said to be fully available without restriction. But this is human data. Does this mean that also detailed personal subject data is available without restriction? Does this respect and guarantee anonymity of the volunteers to a sufficient extent?*
**The Estée Lauder Companies (ELC) have conducted the clinical study during which the samples referred to in this study were collected. ELC have provided an Ethics statement on this study to the editor of PlosOne during the first manuscript submission. In short, in the present study submitted for publication, no product was involved, and the study employed a non-invasive swabbing technique using well established procedures to collect samples for microbiological analysis. Informed consent conforming to ICH GCP standards was obtained from participants prior to participating in the study. All samples collected were anonymized and analysed data cannot be traced back to individual subjects. Apart from the sequencing data, the only data that will be shared are the age group (‘Young’ or ‘Old’) and the derived Skin Aging score (S2 dataset file, first tab).**

*5) The figure quality can be substantially improved but I trust this is done in the next rounds.*
**We expect this is due to the PDF conversion of the file uploads in the submission system. We have ensured that figures are provided that fit the PlosOne guidelines for Tables and Figures.**

*60: Kim et al. (year)?* **added**
*66: Veilonella -> Veillonella?* **Corrected, thank you**
*123: "S1 Table" -> Table S1 (?)* **This is stated as ‘S1 Table’ according to the PLOS ONE's style requirements: “*Format Supporting Information Citations as “S1 Fig”, “S1 Table”, etc*”**
*233: "analogous to PICRUST" -> why not just state the name of this method itself? I am not sure if we should take any specific toolkit as a standard in this field*
**The specific step where we match 16S rRNA to reference genomes is essentially as in PICRUSt, but we use a different database of reference genomes. That is why we used the term “analogous” rather than stating that we use PICRUSt as is. The newly added Figure 1 shows the details of our workflow.**
*391: remove the word "important" and let the readers evaluate the importance.* **Now removed**

**Reviewer #2**

*1) The manuscript is, to some extent exploiting. a circular argument: literature is mined to identify genes, bacterial genomes and host metabolic functions associated to skin aging. Then a data set of microbiome samples from old and young females is analysed to verify that this indeed the case.*
*While this approach has merit, it is difficult to see how this approach could be used to generate new hypothesis. This part in the discussion should be further expanded*.

**We agree with the reviewer that the approach may seem circular. However, the following should be noted: We identified from the literature all the human pathways that were related to skin aging (not necessarily related to microbiome studies) and for which metabolites were also present in molecular pathways of skin commensals. This does not necessarily imply that all bacterial pathways are also operational on the skin, or active in such a way that they actually influence the process of skin aging. This is why the experimental approach with the 16S rRNA gene sequencing from swab samples taken from human volunteers was taken, i.e. to see which of the potential bacterial pathways identified in the first step of the process are in fact active on the skin and are related to skin aging parameters. As such, a new hypothesis for the intersection between host and microbial pathways can be derived, allowing targeted verification in wet-lab studies. This also relates to the comments of the reviewer on (original) Figure 5 (now Fig 6) in which the suggestion is made that all pathways are equally important on the skin.**

**To address this reviewer’s comment, we have included the above-mentioned explanation in the Results section describing (original) Figure 5 (now Fig 6) (Lines 397 – 400 of the ‘revised Manuscript with Track Changes’ file), and in the final paragraph of the Discussion section (Lines 463 – 472 of the ‘revised Manuscript with Track Changes’ file). In addition, we provide an additional statistical analysis as shown in supplementary Table 2 (S2 Table), that not all bacterial pathways contribute equally to skin aging (also see details in our response to this reviewer’s comment below on (original) Figure 5).**

*2) Hidden Markov Models: The authors present the generated collection of HMMs as a useful tool for further research. However this collection is not presented or described in any way (I was expecting to find some link to this collecton, but I have not been able to find it). These should be presented and discussed.*
**In response to this comment, we have now included a schematic representation of the workflow applied in this study, including reference genomes used. In addition, we provided two additional supplementary text files (S1 and S2 Dataset) containing the HMM collection. With these data provided, we believe that anybody skilled in the art will be able to reproduce this workflow.**

*3) Hidden Markov Models: The authors built a collection of bacterial genes (presented in table 1) through literature mining. From this collection of genes and the genome sequences of bacterial strains associated to skin aging they built Hidden Markov Models through multiple sequence alignments, ortholog search and the use of hmmer. While this seems a sensible approach I really don't understand why they don't use the already existing signatures that can be found in databases such as PFAM, Interpro, TIGRFAM and the like. The advantages of using these standardized signatures are obvious as they would provided a more consistent set of identifiers that would enable comparisons between and across datasets . Also the signatures on these databases have been built by considering a more extended collections of genomes should they could be of more use if different species/genera are to be analysed. The authors need to justify why they chose to disregard the already existing databases of standardized signatures.*

**We did not use the standard HMMs in publicly available databases because they are designed to capture a broad range of sequences. These are based on a generic and broad range of microorganisms. The HMMs designed in our study are more specific because they have been created for a narrower set of skin organisms. This of course limits the applications of these models to this specific application. We have included a sentence explaining this in the M&M section (Lines 136 – 138 of the ‘revised Manuscript with Track Changes’ file) of the revised manuscript.**

*4) Validation on skin derived and gut samples: Figure 2 compares the occurrence of these motifs in skin derived and gut derived microbiome samples. However the results don't clearly show these motifs to be more often present on the skin samples or in the gut samples. Analysis of Figure 2 shows that these motifs are also found in the gut microbiome. The authors should explain better to what extent Figure 2 represents a validation of the approach. Also the skin samples in Figure 2 don't seem to be specifically related to aging, so the question is to what extent they expect to have more occurrence of these motifs in these samples.*

**We agree that “validate” is not the correct term to use here; we applied the HMMs to metagenomes from both skin and gut to qualitatively illustrate the differences in abundance of the various models in these two niches. We have therefore rewritten the description of this activity to ‘assess the abundance of the identified bacterial genes in the pathways’ in the Results section in the revised manuscript (Lines 317 – 319 of the ‘revised Manuscript with Track Changes’ file). Additionally, we adjusted the subtitle of the description of this activity in the M&M section to ‘Relative abundance of bacterial functionalities associated with skin aging’.**

*5) Figure 5 shows the link between the pathway level scores and the SA score however it seems that is only for the younger individuals. There seems to be some link, however more exhaustive anlaysis should be presented. One of the main questions is if there is a significant difference in these pathway level scores between individuals with low or high SA. Maybe a t-test or a correlation analysis would provide convincing evidence that would complement the plot on Figure 5. Also it is not clear why the analysis for the older individuals is not presented.*

**We thank the reviewer for this excellent suggestion. We have performed an additional statistical analysis in which for each of the pathways the significance of the correlation between the SA score and the pathway scores was checked by creating a linear model for the relation between the SA score and the pathway score for the individual age groups. The results of this analysis, including a description of the statistical analysis performed, are now provided in an additional supplementary file (S2 Table).**

**The results of this additional analysis indicate that only for subjects of the young age group a significant relation between the SA score was found for the pathways Fatty Acid Biosynthesis, Histidine Metabolism and Radical Protection. We have included the description of these findings in the body text of the Results section, with a reference to Supplementary Table 2 (Lines 403 – 409 of the ‘revised Manuscript with Track Changes’ file).**

*6) I find Appendix S2 to be very informative as it provides a nice review of literature findings. Table S1 provides the list of reference genomes used for this task. It would become more informative if the genes/functions reported in literature that made the authors select each genome were also be reported.*

**In response to this reviewer’s comment, we have now provided the underlying data to Figure 2 as supplementary file (S2 Dataset).**
