# Supplementary material for: Charting host-microbe co-metabolism in skin aging and application to metagenomics data
Source: PLoS One. 2021 Nov 10;16(11):e0258960. doi: 10.1371/journal.pone.0258960 (PMC8580226; doi:10.1371/journal.pone.0258960)
Supplement: Attachment — Submitted filename: Response to reviewers.docx [file pone.0258960.s009.docx]

**Point-to-point response to reviewers’ comments**

**Reviewer #1**

Source code for the experiments has not been provided. Sharing the source code is generally recommended as good practice in bioinformatics research. It is a common misconception that sharing source code equals to delivering a ready-made software product. These are two different concepts, source code for the experiments can be provided "as is", in the form that they were when performing the analyses. If important part of the analysis were conducted manually, and are not documented as source code, the reproducibility and replicability of the analyses is severely limited. The source code should be shared unless there are binding justifications for not doing so. The lack of source code prevents the further evaluation of the implementation reliability, accuracy, robustness, and reproducibility.

**Author’s response**

We further looked into this matter and we’ve now provided a “workflow description” that we deposited in a github repository <https://github.com/andreiprodan/mask-publication> together with the python script (source code) and with reference to the publicly available tools, that were used to generate the HMMs as described in the manuscript. The HMMs can also be downloaded from the Github repository. In addition, we made available the R-script for generating the heatmap in Figure 6 and S2 Table. We trust that making these files publicly available sufficiently covers this reviewer’s comment.

**Reviewer #2**

The authors have addressed all my comments. However an important part of my comment pertains the availability of additional data describing the HMMs and the input files. These files are mentioned in the description of the supporting information but are not available for download. Sometimes submission systems don't easily allow this type of supplementary files. If that is the case, I suggest the authors deposit them in some repository (such as gitlab or github) and include the link.

**Author’s response**

The HMMs and input files were indeed made available in the supplementary datasets that should be available for download. We’ve adopted the suggestion to setup a Github repository and made the HMMs, source code and a description of the workflow available here: <https://github.com/andreiprodan/mask-publication>

The URL of the Github repository is now mentioned in the materials and methods section. The HMMs will also remain available from “S1 dataset. Raw data HMM scripts” in the supplementary materials.
